# 2025 Acknowledgment of *mBio Ad Hoc* Reviewers

**DOI:** 10.1128/mbio.03794-25

**Published:** 2026-02-11

**Authors:** Marvin Whiteley

**Affiliations:** 1 Georgia Institute of Technology 1372, Atlanta, Georgia, USA

## EDITORIAL

To all those who served as reviewers for *mBio* from December 2024 through November 2025, thank you for your outstanding service to the journal. Your thoughtful and timely reviews are the foundation of the journal’s scientific excellence, and I am deeply grateful for the expertise and care you bring to every evaluation.

As I step into the role of editor in chief, I want to express my sincere appreciation for the critical role you play in shaping the quality and impact of the science we publish. I look forward to working closely with you in the coming years as we continue to strengthen *mBio*, elevate the visibility of cutting-edge microbiology, and build an editorial culture rooted in excellence, integrity, and scientific rigor. Your contributions make *mBio* what it is, and I am excited for the future.

Firouz Abbasian

Jeffrey Abbott

Rhea Garces Abisado-Duque

Rachy Abraham

Robert B. Abramovitch

Jacqueline Abranches

Sabrina Absalon

Michael C. Abt

Basel H. Abuaita

Yousef Abu Kwaik

Eisuke Adachi

Mark D. Adams

Philip P. Adams

Walter Adams

Zach Adelman

Herve Agaisse

Surya D. Aggarwal

Jesus Aguirre

Iqbal Ahmad

Waseem Ahmed

Umesh Ahuja

Christopher Aiken

Cecile Alanio

Amelina Albornoz

Patrícia Albuquerque

Laura Alcazar-Fuoli

John F. Alcorn

Mayke Alencar

Gladys Alexandre

Juan Alfonzo

Nazek Al-Gallas

Holly M. Scott Algood

Samuel Alizon

Emma Allen-Vercoe

Thorsten Allers

Timothy Allison

Jontana Allkja

Luke P. Allsopp

Covadonga Alonso

Francis Alonzo

Natalie Saber Al-Otaibi

Adit B. Alreja

J. Andrew Alspaugh

Samuel Alvarez-Arguedas

Nidia Alvarez-Rueda

Lysangela Ronalte Alves

Daniel Amador-Noguez

Gaya Amarasinghe

Carmen Amaro

Zandrea Ambrose

Jorge Amich

Ganesh Anand

Cheryl P. Andam

Christopher J. Anderson

Davide Angeletti

Caetano Antunes

Daniel Aridgides

Ludmilla Aristilde

Robert A. Arkowitz

Catherine R. Armbruster

Chelsie Elizabeth Armbruster

Benjamin Arnold

Gunjan Arora

Gustavo Arrizabalaga

Irina Artsimovitch

Usama Ashraf

John M. Atack

Haruyuki Atomi

Ina Attrée

Walter J. Atwood

Noam Auslander

José L. Avalos

Sandro Azaele

Taj Azarian

Beatriz Eugenia Baca

Jonathan H. Badger

Charles Baer

Frederik Bak

Lauren O. Bakaletz

Brett J. Baker

David A. Baker

Scott E. Baker

Steven F. Baker

Susan C. Baker

Matthew G. Bakker

Elizabeth Balbachevsky

Jose Luis Balcazar

Megan Tierney Baldridge

Mitchell F. Balish

Elizabeth Ballou

David A. Baltrus

Joseph T. Barbieri

James C. A. Bardwell

Rodolphe Barrangou

Michael P. Barrett

Doug Barrick

Anabella A. Barrios

Michael A. Barry

Thomas Bartlett

Christopher F. Basler

Marek Basler

Esmaeil Roohparvar Basmenj

Max Bastian

Rafael Bastos

Tiffany Batarseh

Nidhi Batra

Andrea Battistoni

David L. V. Bauer

Thomas F. Baumert

Andreas J. Bäumler

Simone Becattini

J. David Beckham

Stéphanie Bedhomme

Morgan Beeby

Martin Beer

Peter T. Beernink

Megan G. Behringer

Tiago Beites

Joel G. Belasco

Brittany Jo Belin

Boris R. Belitsky

Deborah Bell-Pedersen

Sandrine Belouzard

George A. Belov

Graham John Belsham

Francesca Benedetti

Jose A. Bengoechea

Lbachir Benmohamed

Richard J. Bennett

Mikkel Bentzon-Tilia

Ivan Berg

Albert M. Berghuis

Cornelia C. Bergmann

Ben Berkhout

Marta Bermejo-Jambrina

Luiz E. Bermudez

Thomas G. Bernhardt

Antonio Bertoletti

Erin M. Bertrand

Sébastien Besteiro

Sumita Bhaduri-McIntosh

Roby P. Bhattacharyya

Zhuyun Bian

Sarah Bigot

Subhash Chandra Bihani

R. Blake Billmyre

André Birgy

Jordan E. Bisanz

William R. Bishai

Alexandre Bisson

David F. Blair

Nicolas Blanchard

Jon S. Blevins

James B. Bliska

Tim R. Blower

Martin Blume

Louis-Marie Bobay

Maksym Bobrovskyy

Paul L. E. Bodelier

Elena Boero

Jens Bohne

Gregory Bokinsky

Paul L. Bollyky

Daniel R. Bond

Joseph Bondy-Denomy

Robert A. Bonomo

Adrianus C. Boon

John C. Boothroyd

Arpita Bose

Guney Boso

Michael John Bottery

Charles Bou-Nader

Robert B. Bourret

Cara C. Boutte

Andrew S. Bowman

John Dallas Boyce

Ian N. Boys

Patricia A. Bradford

Peter J. Bradley

Marc Bramkamp

Alexandra C. Brand

Frederique Braun

Alejandra Bravo

Rachel B. Brem

Benoit Briard

Andrew Bridges

Nicholas Briggs

Volker Briken

Melinda Ann Brindley

Shaun R. Brinsmade

Igor E. Brodsky

Mark Brönstrup

Rebecca M. Brotman

Eric D. Brown

Pamela J. B. Brown

Sam Brown

Edward P. Browne

Hannah Brown Harding

John H. Brumell

Alejandro Brun

Wolfram Brune

Sascha Brunke

Mark P. Brynildsen

Carmen Buchrieser

Nienke Buddelmeijer

Zackery Bulman

Ladislav Bumba

Richard Burchmore

Paul-Christian Burda

Stefan Burén

Alita R. Burmeister

Joshua Burns

Lori L. Burrows

David Burstein

Aisha Burton

Karen Bush

Victor H. Bustamante

Mark J. Buttner

Mariana X. Byndloss

David J. Bzik

Gustavo Caballero Flores

Matthew T. Cabeen

Carlos Caballero

Martin Caffrey

Yuming Cai

Michael J. Calcutt

Benjamin J. Callahan

Andrew Camilli

Joseph Campbell

Isaac Cann

David Cánovas

Pengbo Cao

Yi Cao

Huizhen Cao

Ignazio Carbone

Nicholas H. Carbonetti

Anny Cardenas

Lars-Anders Carlson

Jeremy Carlton

Jason A. Carlyon

Hector Carmona-Salido

Jillian Maree Carr

Christine Carrington

Javier Carrion

Ronan K. Carroll

Vern B. Carruthers

Kayla A. Carter

Agostinho Carvalho

Nicoletta Casartelli

Aitor Casas-Sanchez

Eric Cascales

James E. Cassat

Mariana Castanheira

Santiago Castillo-Ramírez

Nicholas Catanzaro

Roberto Cattaneo

Byron Caughey

Erika Cecon

Jean Celli

Jose P. Ceron-Carrasco

Igor Cestari

Rachna Chaba

Kris Chadee

Arijit Chakraborty

Georgios Chamilos

Patricia A. Champion

Pearl Chan

Ajit Chande

Josephine R. Chandler

Pete Chandrangsu

Wen Chang

Arthur Charles-Orszag

Jeremy Chase Crawford

Soraya Chaturongakul

Anshu Chauhan

Neeraj Chauhan

Diptee Chaulagain

Chen Chen

Dima Chen

James Chen

Joseph Chen

Po-Lin Chen

Qing Chen

Wei Chen

Wenqing Chen

Xiao-Lin Chen

Yi-Wei Chen

Yongzhi Chen

Lei Cheng

Yin-Ru Chiang

Peter Chien

Delma Childers

Christine Chio

Chetan E. Chitnis

Miguel Angel Chiurillo

Petr Chlanda

You-Hee Cho

Katherine Chou

Peter J. Christie

Ewa Chrostek

Nicholas P. Cianciotto

Nicolas Cifuentes-Munoz

Kiera Clayton

Anna R. Cliffe

Cevayir Coban

Bryan Coburn

Alan Cochrane

Charles Cockell

Miguel Cocotl-Yañez

Jörn Coers

Éric A. Cohen

Jean-Francois Collet

Fabian Moritz Commichau

Laurie Comstock

Sarah A. Connolly

Paul J. Converse

James F. Conway

Tyrrell Conway

Gregory Cook

Vaughn S. Cooper

Mirko Cortese

Marco Cosentino Lagomarsino

Vivian Vasconcelos Costa

Elizabeth K. Costello

Peggy A. Cotter

Timothy L. Cover

Lisa Craig

Robert A. Cramer

Samantha Crane

John E. Cronan

Robert W. Cross

Ian Crozier

Laszlo N. Csonka

Valeria C. Culotta

Simon M. Cutting

Joel B. Dacks

Christiane Dahl

Manman Dai

Zachary D. Dalebroux

Ankur B. Dalia

Frédéric Dalle

Julian Damashek

Prashant Purushottam Damke

Ajai A. Dandekar

Jing Dang

Rene Daniel

Pranav Danthi

Sophie E. Darch

Andrew J. Darwin

Dibyadyuti Datta

Matt Daugherty

J. Muse Davis

William G. Davis

Pranav Dawar

Tova Day

Anisha Dayaram

Dennis R. Dean

James A. Decaprio

Valérie De Crécy-Lagard

Miranda De Graaf

Champion Deivanayagam

Ronnie De Jonge

John P. Dekker

Nicholas R. De Lay

Carlos Del Fresno

Victor De Lorenzo

Maurizio Del Poeta

Caroline Demangel

Natalia De Miguel

Marcos H. De Moraes

Christophe D'Enfert

Xin Deng

Xufang Deng

Yinyue Deng

Zengqin Deng

Mark R. Denison

Eric Y. Denkers

Solène Denolly

William H. Depas

Emily Derbyshire

Keith M. Derbyshire

Isabelle Derré

Jeremy P. Derrick

Jigar V. Desai

Mahesh S. Desai

Sanjay A. Desai

Albert Descoteaux

Ulrich Desselberger

Darrell Desveaux

Corrella S. Detweiler

Damien Devos

Rory D. De Vries

Eric Déziel

Eduardo Diaz

Alvaro Díaz

Felipe Diaz-Griffero

Thomas Dick

Allison Louis Didychuk

Diego G. Diel

Binh An Diep

Lars E. P. Dietrich

Stephen Digiuseppe

Jimmy D. Dikeakos

Christine Jade Dilla-Ermita

Joseph P. Dillard

Tatiana Dimitriu

Chan Ding

Qiang Ding

Siyuan Ding

Jocelyne Diruggiero

Dirk P. Dittmer

Armin Djamei

Steven Philip Djordjevic

Ulrich Dobrindt

Roberto Docampo

Yohei Doi

Stephen K. Dolan

Gang Dong

Hai-Sheng Dong

Tao Dong

Xiyang Dong

Sean William Dooling

Sarah E. F. D'Orazio

Robert J. Dorosky

Zhicheng Dou

Florian Douam

Simon L. Dove

Cynthia S. Dowd

Matthew Thomas Doyle

Stephen R. Doyle

Anna Dragoš

Djamel Drider

Arnold J. M. Driessen

Milton T. Drott

Rebecca A. Drummond

Irina S. Druzhinina

Cheng-Guo Duan

Dongsheng Duan

Rebecca M. Dubois

Sarah Dubrac

Yves F. Dufrene

Kenneth Dumack

Samantha L. Durfey

Bryndan Durham

Rebecca Ellis Dutch

Jeffrey D. Dvorin

Michelle Dziejman

Ron Dzikowski

Alison J. Eastman

Gregory D. Ebel

Carrie Eckert

Adrianne N. Edwards

Andrew Edwards

Robert A. Edwards

Monika Ehling-Schulz

Maryna C. Eichelberger

Zehava Eichenbaum

Bart A. Eijkelkamp

Kathy Eisenach

Johanna R. Elfenbein

Melissa Ellermann

Craig D. Ellermeier

Marie A. Elliot

Stéphane Emiliani

Markus Engstler

Christian Engwerda

Luis Enjuanes

Jost Enninga

Gen Enomoto

Marc Erhardt

Joel Ernst

Pedro Escoll

Saber Esmaeili

Ronald Drew Etheridge

Oier Etxebeste

David T. Evans

Heather M. Evans

Benjamin Ezraty

Franziska Faber

Ahmad Fakhoury

Brian A. Fallon

Guo-Chang Fan

Wenxia Fang

Ferric C. Fang

Ariberto Fassati

Herman W. Favoreel

Allison Fay

Na Fei

Michael Feldbrügge

Simon Fellous

Hanping Feng

Li Feng

Pinghui Feng

Ruofei Feng

Youjun Feng

Zhixin Feng

Larissa Fernandes

Lorena Fernandez

Christopher Fernandez-Prada

Rosana B. R. Ferreira

Paul D. Fey

Paul L. Fidel

David A. Fidock

Aretha Fiebig

Kenneth A. Fields

Joshua Fierer

Wendy Figueroa

Martin Filion

Sergio R. Filipe

Gabriel M. Filippelli

Sabine Fillinger

Alain Filloux

Turlough Finan

Steven E. Finkel

Francesco Fiorentino

Anthony John Fischer

Hannah L. Fischer

Skye R. S. Fishbein

James Michael Fleckenstein

Andre Fleissner

Anat Florentin

Enrique Flores Garcia

Josué Flores-Kim

Matthew H. Foley

Suan-Sin Foo

Karl Forchhammer

Adriana Forero

Kevin Forsberg

Jarrod R. Fortwendel

Ron A. M. Fouchier

Slim Fourati

Muinah Adenike Fowora

Elizabeth M. Fozo

Amelie Fradet-Turcotte

Ashwanth Francis

Carolin Frank

David N. Fredricks

Eric O. Freed

Dorte Frees

Johannes Freitag

Nancy E. Freitag

Steven Frese

Eva Frickel

Matthew B. Frieman

Friedrich Frischknecht

Georg Fritz

Julia Frunzke

Katsuya Fuchino

Koh Fujinaga

Kevin Fuller

Kristen Funk

Dana H. Gabuzda

Michaela Ulrike Gack

Attila Gacser

Sarah L. Gaffen

Marta M. Gaglia

Raj Gaji

Kelley Gallagher

Tom Gallagher

Jessica Galloway-Pena

Michael Galperin

Qian Gao

Shou-Jiang Gao

Brandon Garcia

Adolfo García-Sastre

Monica Adriana Garcia-Solache

Susanne Gebhard

Michael J. Gebhardt

Patricia Geesink

Otto Geiger

Tobias Geiger

Cecilia Geijer

Edward Geisinger

Martin Gengenbacher

Georgios Germanidis

Elodie Ghedin

Sourish Ghosh

Sean M. Gibbons

David P. Giedroc

Tim Gilberger

Sabine Gilch

Gillian H. Gile

Michael L. Ginger

Mathieu Gissot

Pierre Gladieux

N. Louise Glass

Erin Gloag

Emile Gluck-Thaler

Joanna B. Goldberg

Marcia Goldberg

Ido Golding

Harris Goldstein

Douglas T. Golenbock

Erin D. Goley

Lisandro Gonzalez

Christopher Dean Goodman

Uri Gophna

Stephen V. Gordon

Kamil Górecki

Andrew Gorringe

Heinrich Georg Gottlinger

Kristie Goughenour

Celia W. Goulding

Mark Goulian

Alexandre Gouzy

Neil A. Gow

Kevin Gozzi

Marcin Grabowicz

Jay Graham

Thomas Gramberg

Stephanie Gras

Juris A. Grasis

Michael Jeffrey Gray

Erin R. Green

David E. Greenberg

Chris Greening

Mark Gresnigt

Anne Grove

Harald Ronald Gruber-Vodicka

Christoph Grundner

Haidong Gu

Wenyu Gu

Eric Guédon

Isis Guibert

Suryaram Gummuluru

John S. Gunn

Chunhe Guo

Guijie Guo

Emily Gurley

James Gurney

Jenna Guthmiller

Maria Hadjifrangiskou

Daniel P. Haeusser

Christine L. Hagan

Rong Hai

George Hajishengallis

Dipasree Hajra

Anders P. Hakansson

Régis Hallez

Brian K. Hammer

Neal D. Hammer

Tobin Hammer

Melanie Hamon

Jun Han

Nam Soo Han

Jo Handelsman

Blake M. Hanson

Ashraful Haque

Hermie J. M. Harmsen

Joe Harrison

Rasika M. Harshey

Dennis J. Hartigan-O'Connor

Caroline S. Harwood

Seyed Ehtesham Hasnain

Roland Hatzenpichler

Vasili Hauryliuk

Christopher S. Hayes

Michelle Hays

Jianguo He

Yong-Xing He

Zhili He

Nicholas S. Heaton

P. Scott Hefty

Hans Heider

Kai Heimel

David E. Heinrichs

Mark T. Heise

Joseph Heitman

Francois Helle

Joris Hemelaar

Andrew J. Henderson

Jeffrey P. Henderson

Rory Henderson

David R. Hendrixson

Carolina Henritta Pohl

Begoña Heras

Paul J. Hergenrother

Jennifer K. Herman

Martina Herrmann

Anat A. Herskovits

Matthias Hess

Volker Heussler

Erik L. Hewlett

Heather Hickman

Ryota Hidese

Stephen Higgs

Hubert Hilbi

Rolf Hilgenfeld

Colin Hill

Jeremy Hirota

Hans H. Hirsch

Kei Hiruma

Joanne K. Hobbs

Craig A. Hodges

Daniel Hoft

Tobias M. Hohl

Matthew T. G. Holden

Caitlyn L. Holmes

Kathryn Elizabeth Holt

David C. Hooper

Stacy M. Horner

Alexander R. Horswill

Benjamin A. Horwitz

Paul A. Hoskisson

Sarzana Hossain

Gaopeng Hou

Kai Hou

P. Lynne Howell

Nina Molin Høyland-Kroghsbo

Hong-Ming Hsu

Fenghua Hu

Junjie Hu

Ke Hu

I-Hsiu Huang

Jun Huang

Kuo-Yang Huang

Lu Huang

Xiaohong Huang

Yao-Wei Huang

Yolanda Huang

Yong Huang

Lori B. Huberman

Karthik Hullahalli

Scott J. Hultgren

Chien-Fu Hung

Peter Hunt

Ryan C. Hunter

Gregory D. D. Hurst

Tuanh N. Huynh

In Young Hwang

Giuseppe Ianiri

Matteo Iannacone

Michael Ibba

Thomas Iftner

Hiroyuki Imachi

James Imlay

Bogdan I. Iorga

Federico Iovino

Keith Ireton

Kelly Ishida

Salim T. Islam

Zahra Fatima Islam

Nuria Izquierdo-Useros

Clay Jackson-Litteken

Timothy James

Eric Jan

Guilhem Janbon

Manon Janet-Maitre

Slavica Janevska

Victoria Jeffers

Urs Jenal

Robert R. Jenq

Ki Jun Jeong

Honglin Jia

Guochun Jiang

Jun jun Jiang

Shibo Jiang

Yan-Mei Jiao

Jose M. Jimenez-Guardeño

Dong-Yan Jin

Peng Jin

Jörgen Johansson

Jeremiah Johnson

John Bernet Johnson

Clare Jolly

Kristina Jonas

Albert Jordan

Joerg Jores

Sarah B. Joseph

Howard S. Judelson

Jae U. Jung

Kirsten Jung

Kwang-Woo Jung

Won Hee Jung

Jennifer Ann Juno

Brandon L. Jutras

Mehdi Kabbage

Jonathan C. Kagan

Manjula Kalia

Sachie Kanatani

Melissa Kane

Congbao Kang

Usheer Kanjee

Ulrike Kappler

John Karijolich

Anna Karnkowska

Kazuyuki Kasahara

Fatah Kashanchi

Justin R. Kaspar

Alexis Kaushansky

Karan G. Kaval

Thomas H. Kawula

Zuzanna Maria Kaźmierczak

Joseph Keane

Jay D. Keasling

Kylene Kehn-Hall

Alison M. Kell

Nancy P. Keller

Frank Kempken

Sean P. Kennedy

Linda J. Kenney

Robyn S. Kent

Rebecca Keogh

Joshua D. Kerkaert

Benjamin Kerr

Sannula Kesavardhana

Lily Khadempour

Denys A. Khaperskyy

Megan R. Kiedrowski

Tammy L. Kielian

Marjolein Kikkert

Hwan Keun Kim

Kami Kim

Minsu Kim

Pyo Kim

Samantha Jane King

Deitsch Kirk

Karla Kirkegaard

Laura A. Kirkman

Morten Kjos

Roland Klassen

Michiel Kleerebezem

Eric A. Klein

Uli Klümper

Lívia Kmetzsch

Dorota Kmiec

David M. Knipe

Katrin Knittel

Leigh Knodler

Bostjan Kobe

Hans-Georg Koch

Matthias Koch

Kamila Kochan

Mattheos A. Koffas

Seiji Kojima

Jay Kolls

Ilana Kolodkin-Gal

Roberto Kolter

James B. Konopka

Anna Konovalova

Michael Koomey

Nicole Marie Koropatkin

Lee Korshoj

Sanna Koskiniemi

Daniel J. Kosman

Yanjun Kou

Markos Koutmous

Caitlin H. Kowalski

Ekaterina Kozaeva

Sven Krapmann

Barry N. Kreiswirth

Tino Krell

Natacha Kremer

Christopher J. Kristich

Abby R. Kroken

Laurie T. Krug

Damian J. Krysan

Karl Kuchler

Richard J. Kuhn

Deepak Kumar

Dilip Kumar

Sanjai Kumar

Rolf Kümmerli

Rainer Kurmayer

Mamuka Kvaratskhelia

Younghwan Kwak

Taejoon Kwon

George Boateng Kyei

Bradley John Laflamme

Michael Lagunoff

Thomas Lahaye

Erh-Min Lai

Yong Lai

Laimonis A. Laimins

Seema Lakdawala

Géraldine Laloux

Rebecca L. Lamason

Noelia Lander

Philippe Langella

Ryan A. Langlois

Luis F. Larrondo

Amel Latifi

Gee W. Lau

Chris Lauber

Raphaël Laurenceau

Matthew B. Lawrenz

Helen M. Lazear

Nina Le Bert

Pierre Lê-Bury

Chia Y. Lee

Jean Claire Lee

Jong-Soo Lee

Min Ja Lee

Pei-Chung Lee

Sunhee Lee

Salome Leibundgut-Landmann

Petr G. Leiman

Silke Leimkühler

Claire Le Hénaff Le Marrec

Katherine P. Lemon

Jose A. Lemos

John M. Leong

Michele Leroux

Christian Lesterlin

David B. Levin

Rodney L. Levine

Stuart M. Levitz

Kim Lewis

Zachary A. Lewis

Ruth Ley

Bin Li

Chunhao Li

Feng Li

Gene-Wei Li

Hongmin Li

Kun Li

Min Li

Su Li

Wentao Li

Xin Li

Yanhua Li

Yize Henry Li

Apostolos Liakopoulos

Jiazhang Lian

Bo Liang

Tami Lieberman

Susan Liebman

James Lightfoot

Dominique H. Limoli

Chi-Jan Lin

Gang Lin

Yong Lin

Paula Lincona-Limon

Richard James Lindsay

Daniel Lingwood

Peter N. Lipke

Gianni Liti

Guanqun Liu

Haoping Liu

Huiquan Liu

Jue Liu

Shan-Lu Liu

Si Liu

Xiaomin Liu

Yongai Liu

Zhengchang Liu

Małgorzata Barbara Łobocka

Frank E. Löffler

Catherine M. Logue

Volker Lohmann

Feng Long

F. Xavier Lopez-Labrador

Marcelo Lopez-Lastra

Michael C. Lorenz

Stilianos Louca

Marta Lourenco

Sebastian Lourido

Ricardo O. Louro

Susan T. Lovett

Adrian Low

Tiffany M. Lowe-Power

Junbo Luan

Aron E. Lukacher

Julius Lukeš

Min-Hua Luo

Zhao-Qin Luo

Antoni Luque

Marina Lusic

Jonathan B. Lynch

Shawn Lyons

Stacy M. Horner

Wenjun Ma

Zhe Ma

Andrew Macdonald

Jason Mackenzie

Erich R. Mackow

Hiten Madhani

Katsumi Maenaka

Martin C. J. Maiden

Andrea Maisner

Joanna Majewska

Johnson Mak

Fabienne Malagnac

Frank Maldarelli

Richard Malley

Jacob Malone

Stanley Maloy

Gideon Mamou

Mark J. Mandel

Balaji Manicassamy

Nicholas J. Mantis

Yuxin Mao

Katrina Mar

John C. March

Antonio Marcilla

Andrés E. Marcoleta

William Margolin

Leonid Margolis

Claudia N. H. Marques

Eric Martens

Benoit Marteyn

Juan F. Martín

Adrian Martineau

Pamela Martinez

Luis Martinez-Sobrido

Willames M. B. S. Martins

Chad Masarweh

Ameya A. Mashruwala

Charles Mason

Paola Massari

Turina Massimo

Anna Karolina Matczuk

Ivan Matic

Miguel A. Matilla

Stephen Matthews

Karen L. Maxwell

Christoph Mayer

Katrin D. Mayer-Barber

Jere W. McBride

Mark J. McBride

Malcolm J. McConville

John K. McCormick

Richard McCulloch

Megan C. McDonald

Sarah McDonald Esstman

James B. McKinlay

James B. McLachlan

Lisa K. McLellan

Katherine McMahon

Case McNamara

Joan Mecsas

Andrew Mehle

Maliheh Mehrshad

Robert Meineke

Keira Melican

John W. Mellors

Didier Menard

Marc Meneghini

Paola E. Mera

Andres Merits

Justin Merritt

Benjamin Meyer

Jinfeng Miao

Miao Miao

Anthony Michael

Charlotte Michaux

Tam Mignot

Laura A. Mike

Aleksandra Milewska

Kingston H.G. Mills

Tohru Minamino

Tiago W. P. Mineo

Lisa Miorin

Aaron P. Mitchell

Angela Marie Mitchell

Valerie Mizrahi

Edward S. Mocarski

Thomas Mock

Axel Mogk

Yasir Mohamud

Ipsita Mohanty

Ian Mohr

Anne Moir

Sachel S. Mok

Bastian Molitor

Jakob von Moltke

Denise M. Monack

Arindam Mondal

Jonathan M. Monk

Penny L. Moore

Nathaniel John Moorman

Danesh Moradigaravand

Diana K. Morales

Dana G. Mordue

Benjamin R. Morehouse

Christopher Morgan

Iain M. Morgan

Eiji Morita

Yasu S. Morita

Robert M. Morris

Joachim Morschhäuser

Lawrence I. Mortin

Heba H. Mostafa

Cristiano Mota

Luís Jaime Mota

Dallas Mould

Bryan C. Mounce

Jorge Moura De Sousa

W. Scott Moye-Rowley

Iwona Mruk

Philip A. Mudd

Frauke Muecksch

Frank Mueller

Elke Mühlberger

Budhaditya Mukherjee

Sampriti Mukherjee

Biswarup Mukhopadhyay

Suchetana Mukhopadhyay

Albert Mulenga

Mandy Muller

Conrad W. Mullineaux

Ulrike G. Munderloh

Joshua Munger

Jose M. Munita

César Muñoz-Fontela

Eric Muraille

Vasant Muralidharan

D. Na

Carey D. Nadell

Shailesh Nair

Beiyan Nan

Vinay Kumar Nandicoori

Arjan Narbad

William Wiley Navarre

Fernando Navarro-Garcia

Debasis Nayak

Stuart J. Neil

Daniel R. Neill

Chris Netherton

Jeniel E. Nett

Benjamin W. Neuman

Donna M. Neumann

Gilles Nevez

Irene L. G. Newton

Wai-Leung Ng

Jordan Ngo

Nguyen Thi Khanh Nhu

Anthony V. Nicola

Rosario Nicoletti

Kirsten Nielsen

Pablo Ivan Nikel

Leonardo Nimrichter

Keita Nishiyama

Estanislao Nistal-Villan

Douglas F. Nixon

Justin R. Nodwell

Cecilia Noecker

Trent Northen

Joshua D. Nosanchuk

Sarah Louise Noton

Estefania Nova-Lamperti

Richard P. Novick

Eric L. Nuermberger

Alejandro Nunez

Catherine O'Connell

Tamara J. O'Connor

Ursula Oggenfuss

Tomoaki Ogino

Adefunke Ogunkanbi

Cheryl Y. M. Okumura

Rich Olson

Michal Olszewski

Michal A. Olszewski

Teresa R. O'Meara

Andrew B. Onderdonk

Luis Humberto Orellana

Carlos J. Orihuela

Kim Orth

Mayuko Osada-Oka

Nir Osherov

Kyla S. Ost

Mike Osta

Lisa Ostereich

Michael T. Osterholm

Mary P. O'Sullivan

Sreekumar Othumpangat

George O'Toole

Karen Otteman

Michael Otto

Morten Otto Alexander Sommer

Daniel E. Otzen

Jon E. Paczkowski

Pranesh Padmanabhan

Gustavo F. Palacios

Massimo Palmarini

Lauren D. Palmer

Tracy Palmer

Timothy Palzkill

Hongyu Pan

Gunjan Pandey

Ivona Pandrea

John C. Panepinto

Mamata Panigrahi

Ralph Panstruga

Kai Papenfort

Coral Pardo-Esté

Alexander R. Paredez

Kristin N. Parent

Donghyun Park

Eunsook Park

Hyunsook Park

Junyoung Park

Yoonkyung Park

Dane Parker

John S. Parker

Raghuveer Parthasarathy

Marcela F. Pasetti

Ranjana Pathania

Atmika Paudel

Ian T. Paulsen

William A. Paxton

Jaclyn S. Pearson

Malik Peiris

Vladimir Pelicic

Daxin Peng

Yousong Peng

Santiago Peralta

Daniel R. Perez

John R. Perfect

David S. Perlin

Steve Perlman

Deborah Perlstein

Christopher Perritore-Galve

Alexandre Persat

Jason Peters

Jason M. Peters

Alexander Petroff

Stephanie Pfaender

Eugen Pfeifer

Derek Pickard

Agnieszka Pietrzyk-Brzezinska

Thomas Pietschmann

Gorben Peter Pijlman

Catarina Pimentel

David S. Pisetsky

Emmanuel Planel

Stefan H. Pöhlmann

Georg Pohnert

Laurent Poirel

Grace Pold

Alessandra Polissi

Simone Pompei

Selvarangan Ponnazhagan

Owen Pornillos

Steven L. Porter

Jan Potempa

Aimee D. Potter

Andrew W. Pountain

Rachel A. Powers

Gabriele Pradel

Nuvee Prapasarakul

B. V. Venkataram Prasad

Lance B. Price

Corinna Probst

José Luiz Proenca-Modena

Amy Pruden

Jude Marek Przyborski

Juan Pu

Tanel Punga

Aaron Webster Puri

Dohun Pyeon

Zhaohui Qian

Udi Qimron

Qi-Long Qin

Baosheng Qiu

Hua-Ji Qiu

Nancy Raab-Traub

Justin D. Radolf

Lilliana Radoshevich

Manuela Raffatellu

Lakshmi Rajagopal

Stuart A. Ralph

Markus Ralser

Katherine S. Ralston

Sanjay Ram

Gordon Ramage

Chandra Ramakrishnan

Kumaran S. Ramamurthi

Marie-Anne Rameix-Welti

Hugo Ramírez-Saad

Sephra Nalini Rampersad

Matthew Ramsey

Anmoldeep Randhawa

Tara M. Randis

Chad A. Rappleye

Philip N. Rather

Lee Ratner

John F. Rawls

Patrick C. Reading

Katarina Rebrosova

Vijay S. Reddy

Matthew Reeves

Aaron Regberg

Neta Regev-Rudzki

E. Hesper Rego

Juan Reguera

Aaron W. Reinke

David Rekosh

Rajko Reljic

Jaanus Remme

Yi Ren

Olaya Rendueles-Garcia

Laurent Renia

Christopher Rensing

Felix A. Rey

Kyu Young Rhee

Johanna Rhodes

Ezio Ricca

Andrew Rice

Peter A. Rice

Anthony R. Richardson

Shannon Esher Righi

Filipa Rijo-Ferreira

Rita Vitorino Moreira Rio

Sebastián Riquelme

Douglas Risser

Jennifer M. Ritchie

Simon K.-M. R. Rittmann

Amariliz Rivera

Juan Rivera-Correa

Erle S. Robertson

Pamela Elizabeth Rodríguez

Elias Rodriguez Olivera

Andrew J. Roe

P. David Rogers

Pejman Rohani

Sanjay K. Rohaun

Robin Rebecca Rohwer

Francisco J. Roig

Enrique Rojas

Fernando Rojo

Antonis Rokas

Ronda J. Rolfes

Jeffrey A. Rollins

Jesus A. Romo

Carlotta Ronda

Severin Ronneau

Jessica Rooke

R. Martin Roop

Michael J. Root

Jason W. Rosch

Alex Rosenberg

Amy Beth Rosenfeld

Emily E. Rosowski

Brittany Ross

Ted M. Ross

Gian Maria Rossolini

Amelia-Elena Rotaru

Stefan Rothenburg

Sarah Elizabeth Rowe

Daniel Rozen

Zhi Ruan

Maxim Rubin Blum

Tracy Ruckwardt

David Z. Rudner

Natividad Ruiz

Alistair Russell

Edward T. Ryan

Kathleen R. Ryan

Brent J. Ryckman

David L. Sacks

Soumok Sadhu

Marat R. Sadykov

Mohsan Saeed

Saveez Saffarian

Martin Safo

Abhik Saha

Senjuti Saha

Takemasa Sakaguchi

Wilmara Salgado

Irene Salinas

Dor Salomon

Susannah J. Salter

Hubert Salvail

Charles E. Samuel

Johan K. Sandberg

Maria Sandkvist

Joseph E. Sanfilippo

Lamba Omar Sangaré

Dominique Sanglard

Kris Sankaran

Sarah E. Sansom

Felipe H. Santiago-Tirado

Gabriela Santos-Gomes

Pierre Santucci

Sumana Sanyal

Mariana Gabriela Sartorio

Christopher Sassetti

Karla J. F. Satchell

John-Demian Sauer

Uwe Sauer

Saumya Saurabh

David F. Savage

Bevan Sawatsky

Dino Lorenzo Sbardellati

Dave J. Scanlan

Joy Scaria

Gabriel Schaaf

Adam Schaenzer

Dirk-Jan Scheffers

Michael Schindler

Christa M. Schleper

Susan Schlimpert

Amy K. Schmid

Thomas M. Schmidt

Kristopher Schmit

Lutz Schmitt

Mirco Schmolke

Dirk Schnappinger

John Schoggins

Robert T. Schooley

Sabrina Schreiner

Bjoern O. Schroeder

Daniel Schultz

Michael J. Schurr

Martin Schuster

Olivier Schwartz

Jessica A. Scoffield

Rona S. Scott

Jessica C. Seeliger

Cecile Segonzac

Julie A. Segre

Michael Seidl

H. Steven Seifert

Ryan F. Seipke

Adnane Sellam

Bert L. Semler

Anna K. Serquiña

David Joseph Sexton

Mohammad R. Seyedsayamdost

Priya S. Shah

Kathy Ho Yen Shair

Ashu Shamar

Yingli Shang

Yiming Shao

Nathaniel Sharp

Dana K. Shaw

Lindsey N. Shaw

Qunxin She

Timothy P. Sheahan

Lilach Sheiner

Aimee Shen

Bang Shen

Xihui Shen

Zeyang Shen

Zhangqi Shen

Xia-Fang Sheng

Kelly M. Shepardson

Nathan M. Sherer

Lanbo Shi

Zheng-Li Shi

Lei Shi

Naoki Shigi

Takashi Shiratori

Anton M. Sholukh

William C. Shropshire

Joshua D. Shrout

Deepak Shukla

Iart Luca Shytaj

L. David Sibley

Chandni Sidhu

Nikolai Siemens

Paul Sigala

Jonathan J. Silberg

Thomas J. Silhavy

Sónia Silva

Lynn L. Silver

Jared A. Silverman

Olivier Silvie

Meinhard Simon

Francesco Simonetti

Brent W. Simpson

Kobi J. Simpson-Lavy

Rakesh Singh

Ramandeep Singh

Varsha Singh

Photini Sinnis

David A. Six

Barbara S. Sixt

Eric P. Skaar

James M. Slauch

Kornelia Smalla

Amber Smith

Geoffrey L. Smith

Steph Smith

Eric J. Snijder

Morgan S. Sobol

Thierry Soldati

Erica Sonnenburg

Lynn Soong

Herman Spaink

Lauren Speare

Paul Spearman

Brady L. Spencer

Alfred Michael Spormann

Georgia Squyres

Shiranee Sriskandan

Eric V. Stabb

Jason E. Stajich

Sarah Stanley

Nicola R. Stanley-Wall

Kenneth A. Stapleford

Spyridon Stavrou

Laura St. Clair

Benjamin J. Stein

Falko Steinbach

Madison Elaine Stellfox

Ioannis Stergiopoulos

Scott Stibitz

Peter Stief

Ann Stock

Peter Stockly

Daniel Stoebel

Ana Maria Stoian

Gisela Storz

Michael Ray Strand

Katerina Strati

Boris Streipen

Reed M. Stubbendieck

Jörg Stülke

Chun-Li Su

Xin-Zhuan Su

Kanta Subbarao

Karthik Subramanian

Agathe Subtil

Gurol Suel

David J. Sullivan

William J. Sullivan, Jr.

Chongkui Sun

Honglei Sun

Jim Sun

Keer Sun

Xingmin Sun

Yan Sun

Yingjie Sun

Jack Sunter

Mehul S. Suthar

Colin J. Sutherland

Elena Suvorova

Lennart Svensson

Michele S. Swanson

Marc Swidergall

W. Edward Swords

David Sychantha

Robert T. Schooley

Shuhei Taguwa

Wanbo Tai

Christine Tait-Burkard

Ralf Takors

Rita Tamayo

Wenjie Tan

Tomohisa Sebastian Tanabe

Biao Tang

Gong-Li Tang

Liudi Tang

Qiang Tang

Qiyi Tang

Xiaoyu Tang

Rick L. Tarleton

Masato Tashiro

Tiffany Taylor

Lesly A. Temesvari

Italo Tempera

Andreas P. Teske

Nicolas Tessandier

Aartjan J. W. te Velthuis

Wai-Hong Tham

Martin Thanbichler

Preethi Narayani Thattai Ragunathan

Volker Thiel

Chang-Fu Tian

Scott A. Tibbetts

Anna D. Tischler

Alexander Titz

Trevor Tivey

David M. Tobin

Jonathan D. Todd

Richard B. Todd

Tadakimi Tomita

Bruce E. Torbett

Jordi B. Torrelles

Alfredo G. Torres

Paula Traktman

Pier-Luc Tremblay

Shashank Tripathi

Que Chi Truong-Bolduc

Joanna Trylska

Chih-Hsuan Tsai

Kevin Tsai

David C. Tscharke

Long Ping Victor Tse

George C. Tsokos

Renée M. Tsolis

Takafumi Tsuboi

Elaine I. Tuomanen

Massimo Turina

Sondra Turjeman

Aaron Paul Turkewitz

Claire Elizabeth Turner

Joanne Turner

David Turra

Rodney K. Tweten

Kevin M. Tyler

Klaus Überla

Aayushi Uberoi

Tsuyoshi Uehara

William Underwood

Jason W. Upton

Constantin F. Urban

Andrew S. Urquhart

Noriko Urushibara

Lucas J. Ustick

Akhil B. Vaidya

Parag Vaishampayan

Ady Vaknin

Raphael Valdivia

Martina Valentini

Agustin Valenzuela-Fernández

Claudio Valverde

Françoise Van Bambeke

Russell Vance

Philippe E. Van Den Steen

Gisou Van Der Goot

Abigail Vanderheiden

Charles Van Der Henst

Lia Van Der Hoek

Carin K. Vanderpool

Patrick Van Dijck

Jan Maarten Van Dijl

Linda F. Van Dyk

Jan Van Kan

Julia C. Van Kessel

Stefano Vanni

Christiaan Van Ooij

Madeleine Van Oppen

Jan-Peter Van Pijkeren

Norman Van Rhijn

Gilles P. Van Wezel

Jose Vargas-Muniz

Arvind Varsani

Tommi Vatanen

Andres Vazquez-Torres

Anthony G. Vecchiarelli

Tony Velkov

Vittorio Venturi

Abhishek Verma

Laura Veschetti

Karen L. Visick

Ganesh Viswanathan

Anastasia N. Vlasova

Sarah Vogel

Stefanie N. Vogel

Megan B. Vogt

Eberhard Voit

Waldemar Vollmer

Jay Vornhagen

Jovanka M. Voyich

Stéphane Vuilleumier

Diana Lia Vullo

Jatin M. Vyas

Crista B. Wadsworth

Seth T. Walk

Graham C. Walker

Mark J. Walker

Daniel Wall

David A. Walsh

Shanquan Wang

Baozhan Wang

Chengshu Wang

Fengping Wang

Gang Wang

Guangwen Wang

Huabing Wang

Jigang Wang

Jue D. Wang

Junjun Wang

Linqi Wang

Penghua Wang

Qiuhong Wang

Xian-Wei Wang

Xin-Ru Wang

Yen-Wen Wang

Yi Wang

Yu Wang

Brian M. Ward

Thomas Ward

Taylor Ware

Matthew J. Wargo

Digby F. Warner

Christopher M. Waters

Kylie J. Watts

Sing-Sing Way

Tilmann Weber

Wei-Li Wei-Li

Jeffrey N. Weiser

David S. Weiss

Susan R. Weiss

Joshua S. Weitz

Matthew D. Weitzman

Rodney A. Welch

Melanie Wellington

Volker F. Wendisch

Carolin Wendling

Vincent Were

Fiona Whelan

Briana Whitaker

Judith M. White

Theodore C. White

Kevin Whitely

Stephanie Widder

Giovanni Widmer

Matthew S. Wiebe

Blake Wiedenheft

Matthias Wietz

Annegret Wilde

Craig B. Wilen

Travis Wiles

Doris Wilflingseder

Lucas Wilken

Ronnie G. Willaert

Julia Willett

Alexandria Williams

John V. Williams

Rohan B. H. Williams

Kim C. Williamson

Peter R. Williamson

Duncan Wilson

Emma H. Wilson

Rachel Wilson

Sam J. Wilson

Jeffrey Wilusz

Malcolm E. Winkler

Wade C. Winkler

Sebastian E. Winter

Elizabeth Winzeler

Jeffrey H. Withey

Kenneth H. Wolfe

Matthew C. Wolfgang

Christiane Wolz

Joseph K. Wong

Joshua Wong

Lok-Yin Roy Wong

Craig C. Wood

James Woodgett

Daniel J. Wozniak

Karen L. Wozniak

Rachel A. F. Wozniak

Arielle Woznica

Daniel Wrapp

Chenggang Wu

Chien-Ting Wu

Nicholas C. Wu

Ting-Ting Wu

Yuntao Wu

Sebastian Thomas Wurster

Yuchen Xia

Xin Xiang

Zhenting Xiang

Shaobo Xiao

Shuqi Xiao

Yonghong Xiao

Shengsong Xie

Zhou Xing

Jianping Xu

Jin-Rong Xu

Junyu Xu

Ping Xu

Zeling Xu

Chaoyang Xue

Jing Xue

Puja Yadav

Vikas Yadav

Srujana Samhita S. Yadavalli

Peiguo Yang

Qian Yang

Shihui Yang

Zhaomin Yang

Maosheng Yao

George S. Yap

Nigel Yarlett

Qingsong Ye

Hui-Ling Yen

Daniel Yero

Shayne Yin

Wen-Bing Yin

Xin Yin

Sukhwan Yoon

Peter Young

Hongwei D. Yu

Huibin Yu

Xu G. Yu

Hanna S. Yuan

Sheng Yuan

Wang Yue

Katsuyuki Yui

Muhammad Ammar Zafar

Mostafa Zamanian

Dario S. Zamboni

Tiffany M. Zarrella

Fidel Zavala

Merve S. Zeden

Bing Zhai

Cheng-Cai Zhang

Guangzhi Zhang

Haifeng Zhang

Hao Zhang

Keyu Zhang

Long Zhang

Rong Zhang

Rui Zhang

Ruifu Zhang

Sean X. Zhang

Wenyan Zhang

Wenyi Zhang

Zhanquan Zhang

Zhenhua Zhang

Guang-Hui Zhao

Wei Zhao

Wenran Zhao

Xin-Qing Zhao

Zhe Zhao

Bo Zhao

Kun Zhao

Aihua Zheng

Chunfu Zheng

Haixue Zheng

Xuhui Zheng

Yong-Hui Zheng

Ping Zhong

Fang Fang Zhou

Shuntai Zhou

Tongqing Zhou

Yanrong Zhou

Yong Zhou

Zhichao Zhou

Bibo Zhu

Fanxiu Zhu

Guan Zhu

Kui Zhu

Zixiang Zhu

Joseph M. Ziegelbauer

Gert Zimmer

Tomaž Mark Zorec

Peng Zou

Wolfram R. Zückert

